# Very Late Antigen-4 (α_4_β_1_ Integrin) Targeted PET Imaging of Multiple Myeloma

**DOI:** 10.1371/journal.pone.0055841

**Published:** 2013-02-08

**Authors:** Deepti Soodgupta, Michelle A. Hurchla, Majiong Jiang, Alexander Zheleznyak, Katherine N. Weilbaecher, Carolyn J. Anderson, Michael H. Tomasson, Monica Shokeen

**Affiliations:** 1 Department of Medicine, Washington University, St. Louis, Missouri, United States of America; 2 Department of Chemistry, Washington University, St. Louis, Missouri, United States of America; 3 Mallinckrodt Institute of Radiology, Washington University, St. Louis, Missouri, United States of America; 4 Department of Pharmacology & Chemical Biology, University of Pittsburgh, Pennsylvania, United States of America; NIH, United States of America

## Abstract

Biomedical imaging techniques such as skeletal survey and ^18^F-fluorodeoxyglucose (FDG)/Positron Emission Tomography (PET) are frequently used to diagnose and stage multiple myeloma (MM) patients. However, skeletal survey has limited sensitivity as it can detect osteolytic lesions only after 30–50% cortical bone destruction, and FDG is a marker of cell metabolism that has limited sensitivity for intramedullary lesions in MM. Targeted, and non-invasive novel probes are needed to sensitively and selectively image the unique molecular signatures and cellular processes associated with MM. Very late antigen-4 (VLA-4; also called α_4_β_1_ integrin) is over-expressed on MM cells, and is one of the key mediators of myeloma cell adhesion to the bone marrow (BM) that promotes MM cell trafficking and drug resistance. Here we describe a proof-of-principle, novel molecular imaging strategy for MM tumors using a VLA-4 targeted PET radiopharmaceutical, ^64^Cu-CB-TE1A1P-LLP2A. Cell uptake studies in a VLA-4-positive murine MM cell line, 5TGM1, demonstrated receptor specific uptake (P<0.0001, block vs. non-block). Tissue biodistribution at 2 h of ^64^Cu-CB-TE1A1P-LLP2A in 5TGM1 tumor bearing syngeneic KaLwRij mice demonstrated high radiotracer uptake in the tumor (12±4.5%ID/g), and in the VLA-4 rich organs, spleen (8.8±1.0%ID/g) and marrow (11.6±2.0%ID/g). Small animal PET/CT imaging with ^64^Cu-CB-TE1A1P-LLP2A demonstrated high uptake in the 5TGM1 tumors (SUV 6.6±1.1). There was a 3-fold reduction in the *in vivo* tumor uptake in the presence of blocking agent (2.3±0.4). Additionally, ^64^Cu-CB-TE1A1P-LLP2A demonstrated high binding to the human MM cell line RPMI-8226 that was significantly reduced in the presence of the cold targeting agent. These results provide pre-clinical evidence that VLA-4-targeted imaging using ^64^Cu-CB-TE1A1P-LLP2A is a novel approach to imaging MM tumors.

## Introduction

Multiple myeloma (MM) is the second most commonly diagnosed hematologic cancer characterized by immunoglobulin secreting malignant plasma B-cells [1]. Over the past decade significant advances in our understanding of the biology of MM has led to the development of better therapeutic options and improved disease management [2]. Myeloma arises from post-germinal center B-cells and its pathogenesis involves both acquired intrinsic genetic abnormalities as well as changes to the bone marrow (BM) microenvironment [1], [3]. Interactions between myeloma cells and BM stroma enhance tumor survival [4]. Clinical and pre-clinical data have demonstrated that changes in the expression of adhesion molecules facilitate the dissemination of plasma cells out of the BM, leading to malignant transformation, tumor spreading and immortalization [5]. MM cells thrive on strong cell-receptor mediated interactions with the BM microenvironment [3]_ENREF_10. Consequently, therapeutics targeting tumor-microenvironment interactions are currently being evaluated clinically and pre-clinically [6], [7].

**Figure 1:**
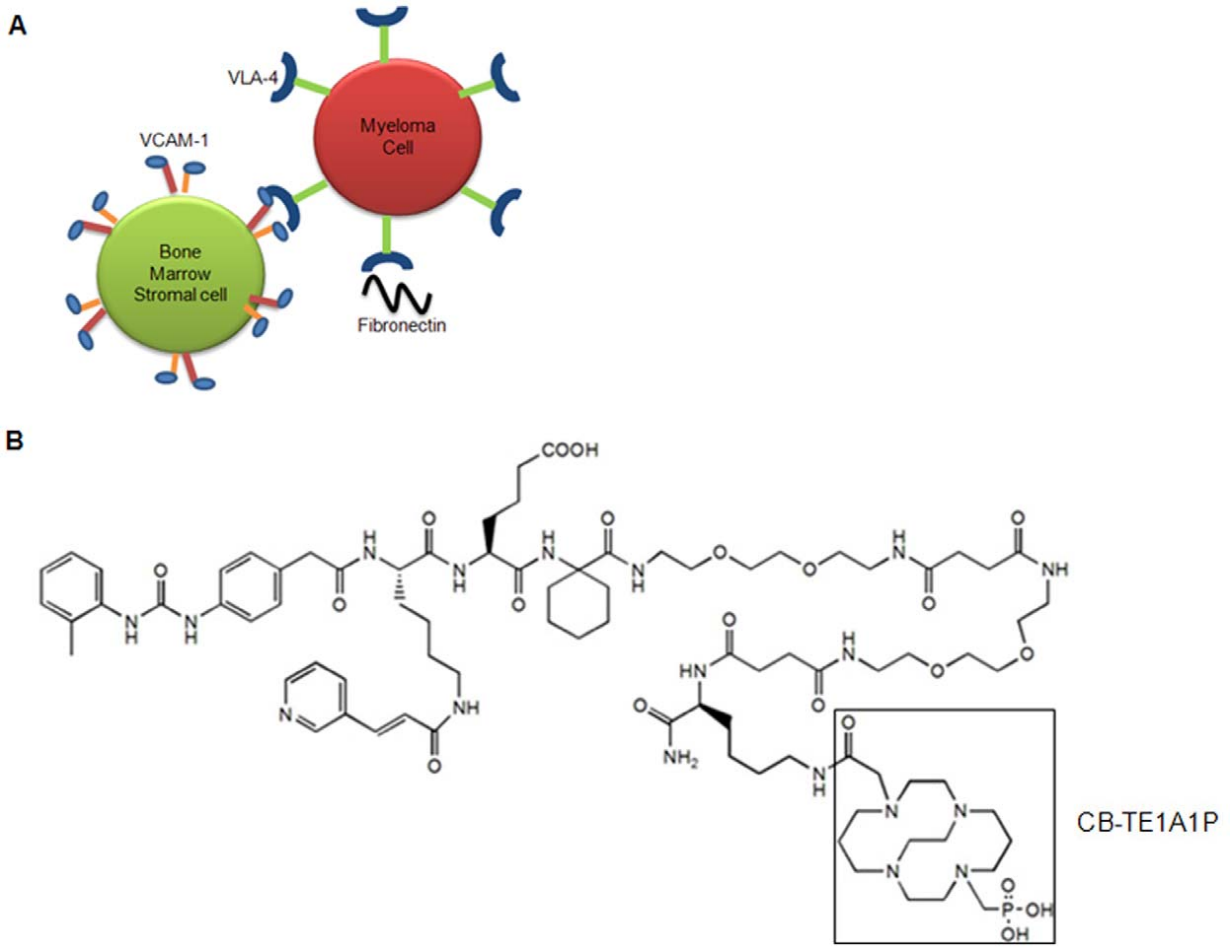
Schematic diagram depicting the interaction of multiple myeloma (MM) and stromal cells. A. Very late antigen-4 (VLA-4, also known as integrin α_4_β_1_) is over expressed on MM cells. VLA-4 mediates myeloma cell adhesion to the bone marrow (BM) stroma through interactions with fibronectin, a glycoprotein of extracellular matrix (ECM) protein and vascular cell adhesion molecule-1 (VCAM-1) protein expressed on bone marrow (BM) stromal cells. B. Structure of CB-TE1A1P-LLP2A.

Very late antigen-4 (VLA-4; also called α_4_β_1_ integrin) is one of the critical mediators of myeloma cell adhesion to the BM stroma (Figure 1A) [8]–[15]. VLA-4 is a non-covalent, heterodimeric, transmembrane receptor that recognizes the QIDS (Gln-Ile-Asp-Ser) and ILDV (Ile-leu-Asp-Val) motifs of two widely known ligands, the vascular cell adhesion molecule-1 (VCAM-1) and fibronection, respectively. It has been demonstrated that in human MM samples, highest detection of plasma cell adhesion molecules was found in patients with active MM [16].

VLA-4 has also been implicated in promoting the activity of bone-resorbing osteoclasts in MM by up-regulating secretion of osteoclast activating factors such as macrophage inflammatory protein (MIP)-1α and MIP-1β [10]. These findings suggest that VLA-4 is a MM marker that is associated with myeloma cell trafficking. Michigami *et al.* used the α_4_β_1_ (VLA-4) positive murine myeloma cell line 5TGM1 and mouse marrow stromal cell line ST2, which expresses VCAM-1, to show that the interaction of myeloma cells with stromal cells *via* α_4_β_1_-integrin/VCAM-1 produces osteoclastogenic activity, suggesting that the presence of stromal cells provide a microenvironment for exclusive colonization of myeloma cells in the BM [12].

VLA-4 also plays an important role in the development of chemotherapy resistance. Noborio-Hatano *et al.* reported that high expression of VLA-4 on the cell surface leads to acquisition of chemotherapy resistance in MM [8]. VLA-4 mediated adhesion and an up-regulated VLA-4 axis is also observed in MM patients who demonstrate chemotherapeutic resistance [17]–[19]. VLA-4, therefore, is a useful marker of tumor cell trafficking, osteoclast stimulation and drug resistance in MM.

Biomedical imaging techniques such as FDG/PET, skeletal survey, bone scintigraphy and MRI are routinely used for staging and post-treatment follow up in MM patients [20]. More importantly, imaging of the skeleton with the aim of detecting lytic bone lesions is needed to discriminate MM from its precursor states such as smoldering MM (sMM) and monoclonal gammopathy of undetermined significance (MGUS) [21]. Radiographic skeletal survey can detect osteolytic lesions only after 30%–50% cortical bone destruction, limiting its sensitivity for imaging early stage myeloma bone lesions. MRI and FDG-PET/CT are comparatively better at detecting bone marrow plasma cell infiltration than conventional radiographs [22]. However, MRI has limitations such as prolonged acquisition time (45–60 min), limiting patient factors such as claustrophobia or metal devices in the body, and particularly, the limited field of view of MRI is not reliable for investigating bones such as skull, clavicle or ribs, and causes frequent under-staging. FDG is a marker of cell metabolism that has limited sensitivity (61%) for intramedullary lesions in MM [23]. Additionally, FDG/PET scan is not recommended within two months following therapy due to high likelihood of healing related (flare phenomenon) false positives. Currently, there are no specific MM imaging agents used clinically. VLA-4 targeted novel molecular imaging of MM has the potential to improve early-stage diagnosis and the management of patients receiving compounds that affect the tumor cells as well as the microenvironment.

Here, we evaluated a VLA-4 targeted PET radiopharmaceutical, ^64^Cu-CB-TE1A1P-LLP2A, (Figure 1B) for PET imaging of VLA-4 positive murine myeloma 5TGM1 MM tumors. For the proof-of-principle imaging studies, we used the 5TGM1 mouse model of bone marrow disseminated mouse MM. The 5TGM1-into-KaLwRij model originates from spontaneously developed MM in aged C57BL/KalwRij mice and has since been propagated by intravenous injection of BM cells from MM bearing mice, into young naive syngeneic recipients [24]. Cell uptake and binding assays performed with 5TGM1 cells demonstrated receptor specific binding of the imaging probe. Tissue biodistribution and small animal PET/CT imaging studies demonstrated highly sensitive and specific uptake of the imaging probe by the subcutaneous (s.c.) and intra-peritoneal (i.p.) 5TGM1 tumors, and suspected tumor cells and associated inflammatory cells in the BM. Additionally, the imaging probe demonstrated high binding to the human MM cells RPMI-8226 *in vitro* that was significantly blocked (P<0.0001) in the presence of the cold targeting ligand. Pilot imaging studies in the orthotopic (intravenous, i.v.) mouse models of mouse (5TGM1) and human (RPMI-8226) MM are ongoing.

## Materials and Methods

### Ethics Statement

All experiments involving the use of radioactive materials at Washington University were conducted under the authorization of the Radiation Safety Committee in accordance with the University’s Nuclear Regulatory Commission license. All animal studies were performed under the Guide for the Care and Use of Laboratory Animals under the auspices of the Washington University Animal Studies Committee. This study was approved by the Washington University Animal Studies Committee (Animal protocol # 20090058).

### Materials

Copper-64 (t_1/2_ = 12.7 h, β^+^; 17.8%, E_β+ max_ = 656 KeV, β^-^, 38.4%, E_β -max_ = 573 KeV) was produced on a CS-15 biomedical cyclotron at Washington University School of Medicine [25]. All chemicals were purchased from Sigma-Aldrich (St. Louis, MO), unless otherwise specified, and solutions were prepared using ultrapure water (18 MΩ-cm resistivity). Radiochemistry reaction progress and purity were monitored by analytical reversed-phase high performance liquid chromatography (HPLC), which was performed on a Waters 600E chromatography system (Milford, MA) with a Waters 991 photodiode array detector and an Ortec Model 661 radioactivity detector (EG&G Instruments, Oak Ridge, TN). An Altima C18 Rocket® column was employed with a gradient that changes from 0.1% TFA in water to 30∶70 0.1%TFA/Water:0.1%TFA/CH_3_CN over the course of 5 min. Radioactive samples were counted using a Beckman 8000 (Franklin Lakes, NJ) automated well-type gamma-counter. PET and CT data were acquired using an Inveon Pre-clinical Imaging Station.

### Synthesis and ^64^Cu Radiolabeling of CB-TE1A1P-LLP2A

CB-TE1A1P was prepared as previously described [26]. Briefly, CB-TE1A1P-LLP2A was designed to have CB-TE1A1P attached to the side chain of Lys and 2 hydrophilic linkers between LLP2A and Lys(CB-TE1A1P). The detailed synthesis of CB-TE1A1P-LLP2A was previously reported [27]. For radiolabeling, Cu-64 chloride (^64^CuCl_2_) (5−10 µL in 0.5 M HCl) was diluted with 0.1 M ammonium acetate buffer (pH 8, 50−100 µL). The CB-TE1A1P-LLP2A solution (5 µg) was diluted with acetate buffer, ^64^Cu-acetate (185 MBq (5 mCi)) was added, and the mixture was incubated at 80–95°C for 5 min or at room temperature for 45–60 minutes. After purification, the radiochemical purity (RCP) of the ^64^Cu-labeled CB-TE1A1P-LLP2A was monitored by radio-HPLC.

### Cell Lines

The human myeloma cell line RPMI-8226 was obtained from the American Type Culture Collection (ATCC). The previously published mouse MM cell line 5TGM1 [28] (a gift from Dr. G. Mundy, Vanderbilt University, Nashville, Tennessee) was grown in DMEM supplemented with 10% fetal bovine serum, penicillin (100 U/ml) and streptomycin (50 mg/ml). Long term culture of the cells occurred in a water jacketed incubator at 37°C and 5% CO_2_. Assays were also carried out under these respective conditions.

### Flow Cytometry

PE-conjugated mAb to mouse CD49d (Integrin alpha 4) was purchased from eBioscience. 5TGM1 cells were prepared for flow cytometry by incubating cells with mAb followed by PBS washes. Data collection and analyses were performed on a FACScalibur flow cytometer (Becton Dickinson Immunocytometry Systems, Mountain View, California, USA).

### *In Vitro* Cell Uptake Assay

Cell uptake assays were performed in murine 5TGM1 and human RPMI-8226 myeloma cells using ^64^Cu-CB-TE1A1P-LLP2A to determine the sum of cell internalized and surface-bound fractions. Cells were grown in Iscoves MDM until 60−75% confluent, harvested by mechanical dissociation, and re-suspended in the binding medium (phosphate buffered saline [PBS], 0.1% bovine serum albumin [BSA] and 1 mM Mn^2+^) in 1.5 mL microfuge tubes. A solution of ^64^Cu-CB-TE1A1P-LLP2A (0.1 nM) was added to the cell suspension. The samples were incubated for 60 min in a cell incubator (37°C, 5% CO_2_). To determine the *in vitro* VLA-4 binding specificity of ^64^Cu-CB-TE1A1P-LLP2A, samples were co-incubated with ∼200-fold excess of the unlabeled ligand, LLP2A. After incubation, the samples were centrifuged at 1,500 rpm for 5 min, and the radioactive medium was removed. Cell pellets were rinsed with ice cold binding buffer (500 µL) and centrifuged at 1,500 rpm for 3 min (2 X). The radioactivity in cell pellets was measured in a well counter (Packard II gamma counter).

### *In Vitro* Saturation Binding Assay

For saturation binding experiments, ^64^Cu-CB-TE1A1P-LLP2A (0.5–15.5 nM) was incubated with ∼250,000 5TGM1 (∼ 0.41 mg protein) whole cells in 1.5 mL microfuge tubes for 2 h at 4°C in a total volume of 500 µL of binding medium (phosphate buffered saline [PBS], 0.1% bovine serum albumin [BSA] and 1 mM Mn^2+^). The reaction tubes were put on a slow moving rotor during the 4°C incubation. After the incubation, samples were centrifuged at 1,500 rpm for 5 min, reaction buffer was removed by vacuum aspiration and the cells were washed two times with ice cold PBS. Non-specific binding was determined by conducting the assay in the presence of an excess (∼200 fold) unlabeled LLP2A. The radioactivity in the cell pellets was measured in a well counter (Packard II gamma counter). The specific binding was obtained by the subtraction of non-specific binding from total binding. The dissociation constant (K*_d_*) and receptor density (B*_max_*) were estimated from the non-linear fitting of the specific binding versus the concentration of ^64^Cu-CB-TE1A1P-LLP2A using Prism software (GraphPad, San Diego, CA).

### Mouse Models of MM

KaLwRij mice (from Dr. Claire M. Edwards, Vanderbilt University Medical Center Cancer Biology, Nashville, TN) were housed in ventilated cage racks and allowed food and water. 5TGM1 cells in log phase growth were prepared for injection by precipitation in a centrifuge followed by a wash step with sterile endotoxin-free PBS. Finally, the cells were re-suspended in endotoxin-free PBS at a concentration of 1×10^6^ cells/mL and injected with or without matrigel, respectively, in the nape of the neck subcutaneously (s.c.) or intraperitoneally (i.p.) in a 100 µL volume in 3–5 week old mice. The tumors were allowed to grow for an average of 10 d.

### Serum Protein Electrophoresis (SPEP) Analysis

Mice were bled by tail grazing at the desired time point. Blood was collected into Microtainer tubes (Becton Dickinson) and was centrifuged for 10 min at 2,300 g. Sera were diluted 1∶2 in normal saline buffer and analyzed by serum protein electrophoresis (SPEP) on a QuickGel Chamber apparatus using pre-casted QuickGels (Helena Laboratories) according to manufacturer’s instruction. Densitometric analysis of the SPEP traces was performed using the clinically certified Helena QuickScan 2000 workstation, allowing a precise quantification of the various serum fractions, including the measurements of gamma/albumin ratio.

### Histological Analysis

After sacrifice from the biodistribution and the small animal imaging studies, the tumor sections were stained with hematoxylin and eosin (H&E) and visualized under a Nikon Eclipse TE300 microscope equipped with a Plan Fluor 20/0.45 objective lens (Nikon) and a Magnafire digital charge-coupled device camera.

### Biodistribution Studies in 5TGM1 Tumor-bearing Mice

5TGM1 tumor bearing mice were sacrificed at 2 or 24 h after the injection of the radiopharmaceutical, ^64^Cu-CB-TE1A1P-LLP2A. Blood, marrow, fat, heart, stomach, intestines, lungs, liver, spleen, kidneys, muscle, bone, pancreas, and tumor were harvested, weighed, and counted in the γ-counter. For the *in vivo* blocking studies, an additional group of mice was injected with the radiopharmaceutical premixed with ∼200-fold excess of LLP2A to serve as a blocking agent and sacrificed at the respective time point. The percent injected dose per gram of tissue (%ID/g) was determined by decay correction of the radiopharmaceutical for each sample normalized to a standard of known weight, which was representative of the injected dose.

### Small Animal Imaging Experiments

Prior to small animal PET/CT imaging, mice were injected intravenously (tail vein) with ^64^Cu-CB-TE1A1P-LLP2A (0.9 MBq (SA: 37 MBq/µg)). At 2 h post injection, mice were anaesthetized with 1–2% isoflurane and imaged with small animal PET (Focus 220 or Inveon (Siemens Medical Solutions, Knoxville,TN)), while the CT images were acquired with the Inveon. Static images were collected for 30 min and co-registered using the Inveon Research Workstation (IRW) software (Siemens Medical Solutions, Knoxville,TN). PET images were re-constructed with the maximum *a posteriori* (MAP) algorithm [29]. The analysis of the small animal PET images was done using the IRW software. Regions of interest (ROI) were selected from PET images using CT anatomical guidelines and the activity associated with them was measured with IRW software. Maximum standard uptake values (SUVs) for both experiments were calculated using SUV = ([nCi/mL]x[animal weight (g)]/[injected dose (nCi)]). A set of mice was also imaged at 24 h post injection.

### Data Analysis and Statistics

All data are presented as mean±SD. For statistical classification, a Student’s t test (two-tailed, unpaired) was used to compare individual datasets. All statistical analyses were performed using Prism software. P values less than 0.05 were considered significant.

## Results

### Radiochemistry and *In Vitro* Studies

Radiochemical purity for ^64^Cu-CB-TE1A1P-LLP2A was >95% as determined by radio-high performance liquid chromatography (radio-HPLC). The specific radioactivity for ^64^Cu-CB-TE1A1P-LLP2A was 37 MBq/µg.

### ^64^Cu-CB-TE1A1P-LLP2A Binding to VLA-4 in 5TGM1 Murine Myeloma Cells

**Figure 2:**
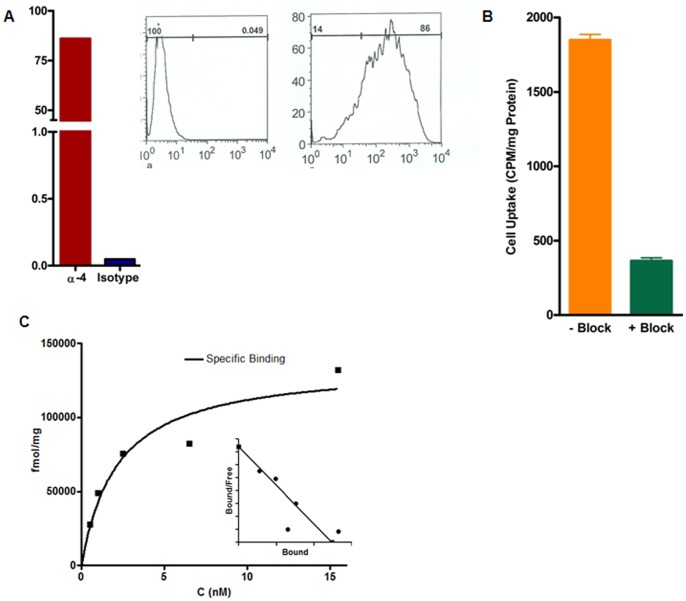
Flow cytometry, cell uptake and saturation binding data. A. Greater than 85% of α_4_ (VLA-4)-positive cells in total 5TGM1 tumor cell population as determined by flow cytometry (Anti-Mouse CD49d (integrin α-4). B. Cell uptake of ^64^Cu-CB-TE1A1P-LLP2A (0.1 nM), in 5TGM1 cells at 37°C (p<0.0001). C. Saturation binding curve for ^64^Cu-CB-TE1A1P-LLP2A gave a K*_d_* of 2.2 nM (±1.0) and B*_max_* of 136 pmol/mg (±19). N = 3 (Inset: Scatchard transformation of saturation binding data).

5TGM1 cells demonstrated high expression (>85% of cells staining positive) of α-4 by flow cytometry when normalized to the isotype control (Figure 2A). The cellular uptake (sum of the cell-internalized and cell surface-bound fractions) at 37°C of ^64^Cu-CB-TE1A1P-LLP2A in 5TGM1 cells in the presence and absence of the blocking agent (non-radiolabeled ligand, LLP2A) was significantly different (p<0.0001, Figure 2B).

The *in vitro* binding affinity of ^64^Cu-CB-TE1A1P-LLP2A was investigated by determining the equilibrium dissociation constant (K*_d_*) and the maximum specific binding (B*_max_*) of the radiolabeled conjugate to 5TGM1 cells in saturation binding assays. A large excess (200-fold excess) of unlabeled LLP2A was added to a parallel set of cells to saturate receptor binding sites and account for non-specific binding. A representative saturation binding curve and Scatchard transformation of ^64^Cu-CB-TE1A1P-LLP2A to 5TGM1 cells is shown in Figure 2C. The data show that in the concentration range of 0.5–15.5 nM, ^64^Cu-CB-TE1A1P-LLP2A is bound to a single class of binding sites with a K*_d_* of 2.2 nM (±0.9) and B_max_ of 136 pmol/mg (±19).

### Biodistribution of ^64^Cu-CB-TE1A1P-LLP2A in 5TGM1 Tumor Bearing Immunocompetent/KaLwRij Mice

**Figure 3:**
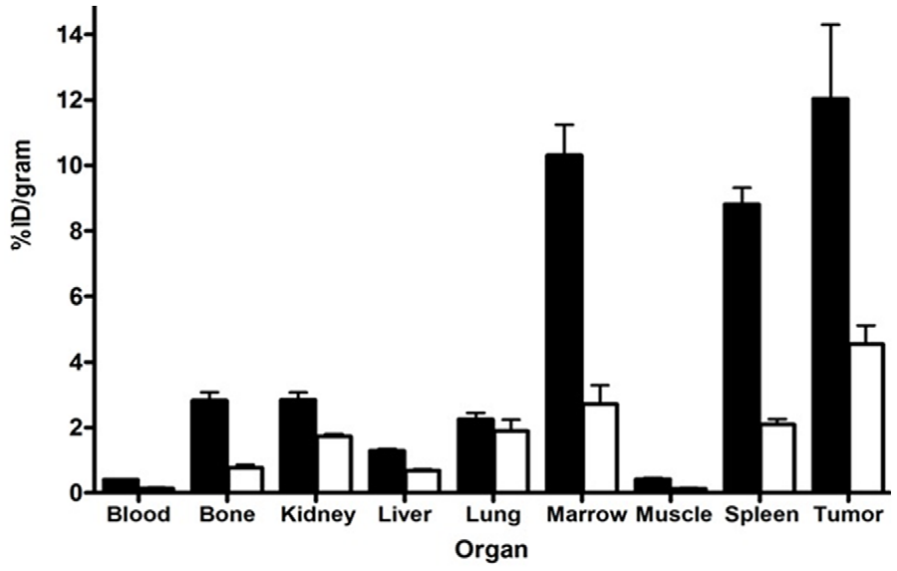
Tissue biodistribution of ^64^Cu-CB-TE1A1P-LLP2A in 5TGM1 s.c. tumor mice. Biodistribution of ^64^Cu-CB-TE1A1P-LLP2A in 5TGM1 s.c. tumor mice (black bars). The open bars represent biodistribution in the presence of non-radioactive blocking agent (∼ 200 fold excess LLP2A). Mice were injected with ^64^Cu-CB-TE1A1P-LLP2A (0.01 µg, 0.2 MBq, SA: 37 MBq/µg) and sacrificed at 2 h post injection. N = 4 mice/group.

*In vivo* biodistribution of ^64^Cu-CB-TE1A1P-LLP2A was evaluated in KaLwRij mice bearing subcutaneous 5TGM1 tumors (Figure 3). Uptake of the radiotracer was high in the 5TGM1 tumors (12.04±4.50%ID/gram). As expected, tracer uptake was highest in the VLA-4 rich hematopoietic organs, spleen (8.8±1.0%ID/gram) and marrow (11.6±2.1% ID/g). In a separate cohort of tumor-bearing mice, excess of cold LLP2A ligand was co-administered with ^64^Cu-CB-TE1A1P-LLP2A. In the presence of the blocking agent, the radiotracer uptake was significantly reduced in the tumor, spleen and bone (p<0.05), demonstrating the *in vivo* binding specificity of ^64^Cu-CB-TE1A1P-LLP2A (Figure 3, open bars). Biodistribution of ^64^Cu-CB-TE1A1P-LLP2A in non-tumor bearing KaLwRij mice was similar to tumor-bearing mice, with spleen and BM being the key uptake organs (data not shown).

### Small Animal Imaging Experiments

**Figure 4:**
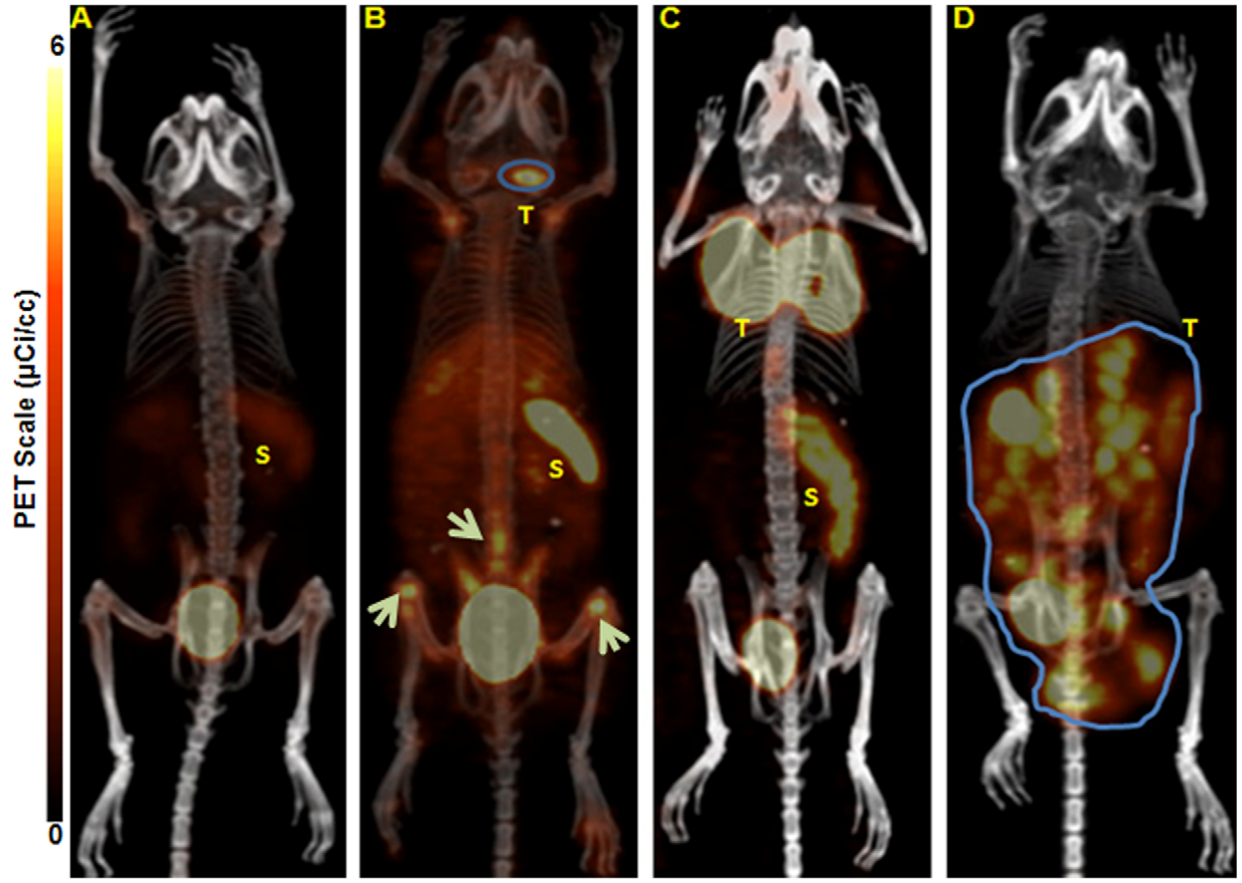
Representative maximum intensity projection (MIP) small animal PET/CT images. A. non-tumor KaLwRij control mouse. B. a small sized, non-palpable, early stage subcutaneous (s.c.) 5TGM1 murine tumor in the nape of the neck inoculated without the use of matrigel (tumor SUV 2.24). White arrows point to suspected tumor cells and associated tumor supporting cells in the BM of the long bones and spine. C. matrigel assisted s.c. 5TGM1 tumor in the nape of the neck (tumor SUV 6.2). D. mouse injected intraperitoneally (i.p.) with 5TGM1 murine myeloma cells. All the mice were injected with ^64^Cu-CB-TE1A1P-LLP2A (0.9 MBq, 0.05 µg, 27 pmol) and were imaged by small animal PET/CT at 2 h post-injection. *All tumor bearing animals were SPEP (Serum Protein Electrophoresis) positive. T = Tumor; S = Spleen. N = 4/group.

**Figure 5:**
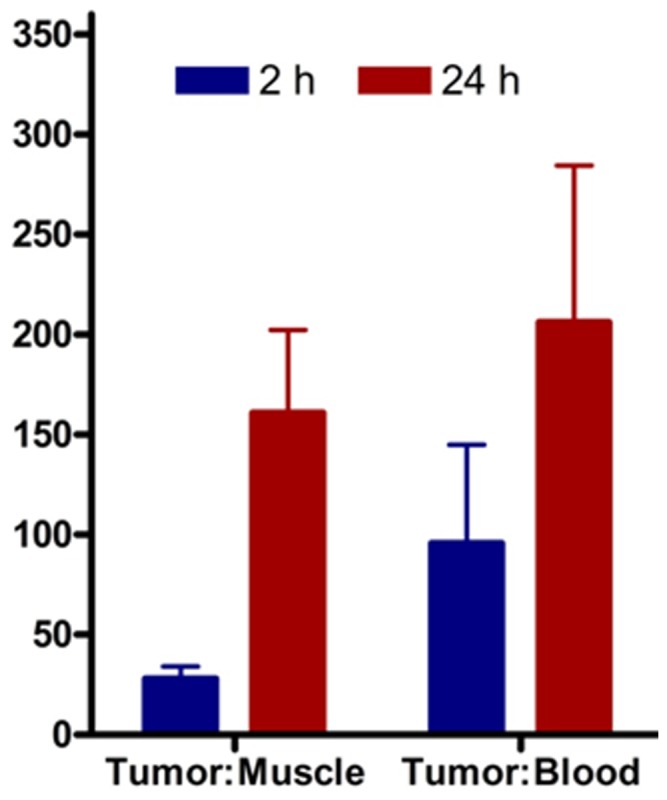
Graph representing tumor to muscle and blood respectively at early and late time-points. The Tumor/Muscle and Tumor/Blood ratios at 2 h and 24 h respectively calculated from the MIP images (SUVs). The ratios were higher at 24 h indicating improved contrast after clearance of the radioactive probe from the background tissues over time.

**Figure 6:**
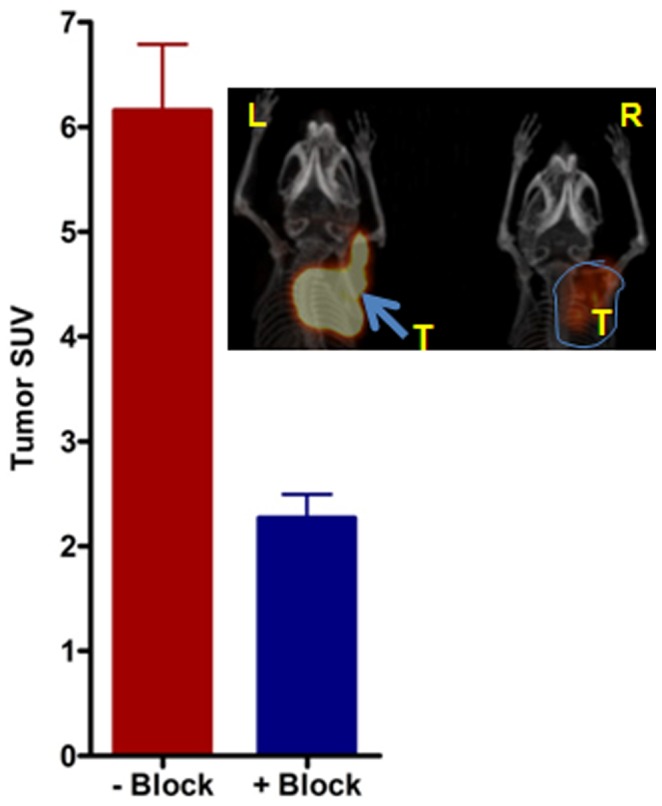
Graph representing *in vivo* blocking of ^64^Cu-CB-TE1A1P-LLP2A. Averaged tumor MIP SUV’s (N = 4) calculated from small animal PET images in the presence and absence of the blocking agent, LLP2A. **Inset**-Representative MIP image showing mice with similar tumors in the nape of the neck imaged in the absence **(L)** and presence **(R)** of the blocking agent (LLP2A). Mice were imaged with small animal PET and ^64^Cu-CB-TE1A1P-LLP2A at 2 h post-injection (0.9 MBq, 0.05 µg, 27 pmol, (SA: 37 MBq/µg). Blocking dose: ∼200 fold excess than the tracer amount. P<0.05.

To test the ability of ^64^Cu-CB-TE1A1P-LLP2A to image MM, small animal PET/CT imaging was conducted in KaLwRij mice bearing 5TGM1 murine myeloma tumors. The following i.p. and s.c. 5TGM1 models were used for the proof-of-principle imaging studies: 1) a non-matrigel assisted s.c. (plasmacytoma) tumor in the flank of the mouse (Figure 4B); 2) a matrigel assisted s.c. tumor in the flank of the mouse (Figure 4C); and 3) tumor cells injected in the peritoneal (i.p.) cavity (Figure 4D). Figure 4 contains four (B-D) representative maximum intensity projection (MIP) small animal PET images using ^64^Cu-CB-TE1A1P-LLP2A (0.9 MBq, 0.05 µg, 27 pmol, SA: 37 MBq/µg) at 2 h post injection in the various models compared to a non-tumor-bearing control mouse (Figure 4A). The small animal PET images with ^64^Cu-CB-TE1A1P-LLP2A demonstrate that the VLA-4 targeted radiopharmaceutical has high sensitivity for detecting myeloma tumors of different sizes and heterogeneity, as even early stage, non-palpable, millimeter sized tumor lesions were clearly imaged (Figure 4B). The SUV of the tumor shown in Figure 4D was not determined due to the large tumor size and overlap with the spleen and bladder. The heterogeneous distribution of the imaging agent in Figure 4D likely corresponds with the heterogeneity of the tumor mass. The uptake of ^64^Cu-CB-TE1A1P-LLP2A in i.p. tumors was determined to be 14.9±2.6%ID/g by post PET biodistribution (2 h post injection). Images collected at 24 h demonstrated significantly improved tumor to background ratios as compared to 2 h (Figure 5). Supplemental image 1 shows a representative small animal PET/CT MIP image of a mouse bearing s.c. 5TGM1 tumor at 2 h and 24 h respectively. The *in vivo* targeting specificity was demonstrated by blocking with excess LLP2A (∼200 fold), which led to reduced uptake in the 5TGM1 MM tumors. As shown in Figure 6, there was a 3-fold (P<0.05) reduction in cumulative tumor SUVs in the presence of the blocking agent (6.2±1.1 vs. 2.3±0.4). A representative MIP image of the reduced tumor uptake is shown in Figure 6 inset. Together, these data demonstrate that ^64^Cu-CB-TE1A1P-LLP2A can be used to image murine MM tumors in a variety of anatomic sites. All the images are scaled the same, demonstrating that although there is uptake in the spleen of a non-tumor bearing mouse (SUV: 2.2), the uptake is higher in the spleens of tumor bearing mice (SUV: 3.3). We are currently investigating the imaging of myeloma induced spleen pathology (splenomegaly) in orthotopic (i.v.) 5TGM1 mouse models of MM.

### Confirmation of 5TGM1 Tumor Burden by Histological and Serum Protein Electrophoresis (SPEP) Analysis

**Figure 7:**
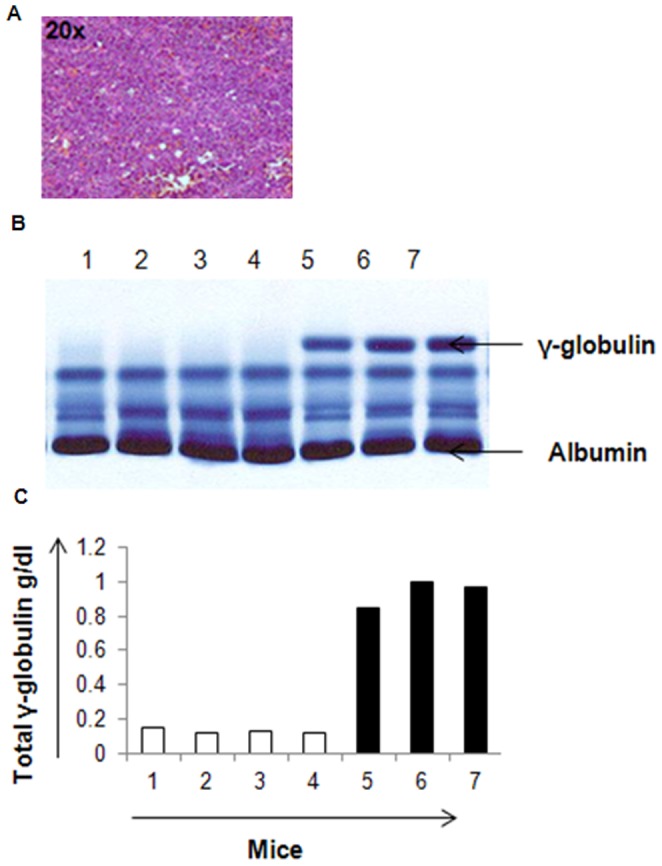
Tumor histology and SPEP (Serum Protein Electrophoresis) analysis on the serum of KaLwRij mice. A. Hematoxylin and eosin (H&E) stained slide of a representative 5TGM1 s.c. tumor tissue. The tumor cells show irregularly shaped nuclei and increased mitosis consistent with myeloma pathogenic features. B. SPEP gel showing qualitatively the γ-globulin (M protein) in tumor bearing (lanes 5, 6 & 7) and non-tumor bearing (lanes 1, 2, 3 & 4) mice. The 5TGM1 tumor bearing mice (lanes 5, 6 & 7) were analyzed two weeks post tumor cell inoculation. The top arrow represents the M Protein band and the lanes represent serum SPEP for each mouse. C) Quantitative representation of the total γ-globulin g/dl in the mice (1–7).

A representative hematoxylin and eosin (H&E) slide of a 5TGM1 s.c. tumor tissue from those imaged in Figure 4 is shown in Figure 7A. The tumor cells show irregularly shaped nuclei and increased mitosis consistent with myeloma pathogenic features. The SPEP test is used clinically to measure clonal γ-globulin (M protein) in the blood to quantify disease burden in MM. SPEP analysis was performed on all tumor-bearing mice. Qualitative and quantitative analyses of the SPEP gels indicated increased M-protein (gamma protein band) in tumor bearing mice as compared to non-tumor control mice.

### Binding of ^64^Cu-CB-TE1A1P-LLP2A to Human RPMI-8226 Myeloma Cells

**Figure 8:**
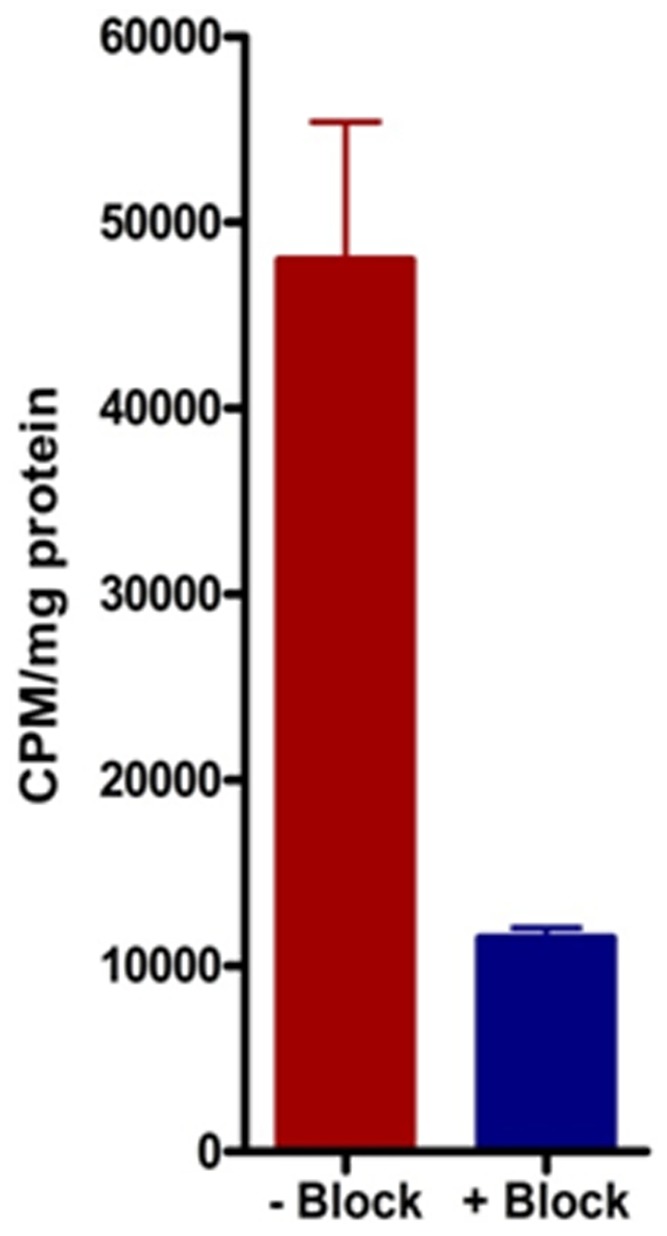
Cellular uptake of ^64^Cu-CB-TE1A1P-LLP2A in human myeloma RPMI-8226 cells. Cell uptake of ^64^Cu-CB-TE1A1P-LLP2A (0.1 nM), in human RPMI-8226 cells at 37°C in the absence (red bar) and presence (blue bar) of excess LLP2A (P<0.0001).

Binding of ^64^Cu-CB-TE1A1P-LLP2A was evaluated in human MM cell line RPMI-8226 *in vitro*. RPMI-8226 cell uptake experiments demonstrated high uptake of ^64^Cu-CB-TE1A1P-LLP2A that was significantly blocked in the presence of excess LLP2A (P<0.0001) (Figure 8).

## Discussion

This study demonstrates novel imaging of MM tumors using a high-affinity, VLA-4 (integrin α_4_β_1_) targeted PET probe, in a s.c. and i.p. (extra-osseous) immunocompetent mouse model of MM.

Clinically, MM is characterized by the presence of end-organ damage such as lytic bone disease, anemia, hypercalcemia, and renal insufficiency [30]. While laboratory tests such as serum protein electrophoresis (SPEP), urine protein electrophoresis, bone marrow evaluation, and serum free light chains are key to making a preliminary diagnosis, molecular imaging assists in locating the tumor lesion within the bone and outside the bone. Imaging of the skeleton, with the aim to rule out lytic bone lesions, is important to discriminate MM from its precursor states. FDG-PET/CT fusion imaging has the ability to detect diffuse or focal lesions in the bone marrow prior to destruction of mineralized bone as well as extramedullary disease in patients [31], [32]. However, FDG-PET/CT is often not effective at imaging subsets of myeloma patients presenting drug resistant (DR) tumors due to intrinsically limited metabolic rates of the DR tumors [33]–[35]. Moreover, FDG-PET/CT also has limitations in the diagnosis or staging of clinically organ-confined or locally recurrent disease since FDG uptake is increased in cells and tissues undergoing rapid division, growth, and inflammatory response. These processes are transient and are not necessarily tumor-specific [36], [37]. VLA-4 based molecular imaging could effectively address these limitations by providing tumor specific information based on receptor status.

In the current study, we evaluated a newly developed mono-acid, mono-phosphonate chelator, CB-TE1A1P, conjugated with a high VLA-4 affinity (IC_50_ = 2 pM) peptidomimetic ligand, LLP2A for PET imaging of MM [27]. LLP2A is comprised of D- and unnatural amino acids which makes it resistant to proteases in human plasma, and has been evaluated as a promising VLA-4 specific therapeutic agent that possesses safety features for potential human use [38]. The conjugate, CB-TE1A1P-LLP2A, can be labeled with Cu-64 in high specific activity under mild conditions [39]. The VLA-4 positive 5TGM1 murine myeloma cells, used for the *in vitro* and *in vivo* studies were an ideal selection for the described proof-of-principle imaging studies [28], [40]. For the animal experiments described in this study, i.p. or s.c. injections of 5TGM1 cells in syngeneic KaLwRij mice led to reproducible extra-osseous tumors. Small animal PET/CT with ^64^Cu-CB-TE1A1P-LLP2A was supported by cell binding studies, SPEP analysis, and *ex-vivo* tissue biodistribution and tumor histology.

The 5TGM1 cell binding of ^64^Cu-CB-TE1A1P-LLP2A was significantly reduced in the presence of excess LLP2A at 37°C and 4°C respectively, signifying receptor mediated endocytosis. The saturation binding assays were performed in the presence of Mn^2+^ ions and produced high B_max_ values (136 pmol/mg (±19)), which is key to a desirable imaging outcome resulting from the high binding potential (B_max_/K_d_) ratio [41]. Cell uptake studies performed in the absence of receptor activating cations, in this case, Mn^2+^, resulted in much lower binding (data not shown). Lam and co-workers have also shown independently that LLP2A binds to the activated form of α_4_β_1_ integrin [38], [42]. Additional studies will be needed to describe the changes in VLA-4 activation status in response to different stimuli [43]. Binding of ^64^Cu-CB-TE1A1P-LLP2A was evaluated in the human MM cell line, RPMI-8226. ^64^Cu-CB-TE1A1P-LLP2A demonstrated high binding to RPMI-8226 cells, which was significantly blocked in the presence of excess LLP2A (Figure 8).

The *ex vivo* biodistribution and *in vivo* imaging studies in the 5TGM1 mouse models of MM support the fact that ^64^Cu-CB-TE1A1P-LLP2A has desirable *in vivo* pharmacokinetics for achieving excellent tumor to background ratios. Three 5TGM1 tumor models were evaluated in this study to demonstrate the versatility of the PET imaging probe. In the mouse bearing s.c. tumor cells without matrigel, PET imagining readily detected the presence of VLA-4 expressing cells localized to the femur, tibia, and spine (Figure 4B, arrows). While this signal is presumed to indicate dissemination of myeloma cells to the bone, further *ex vivo* analyses will be required to characterize the extent that inflammatory or stromal cells may also be imaged as components of the pre-metastatic niche. Regardless, this data demonstrates that VLA-4-targeted PET imaging has the ability to image cells within mineralized bone. *In vivo* tumor blocking of ^64^Cu-CB-TE1A1P-LLP2A was observed in both the biodistribution and small animal PET imaging studies. Small animal PET images collected at 2 h post-injection had high tumor to muscle ratios (28±6) (Figure 5 and supplemental image 1), with improved ratios observed at 24 h time point due to the clearance from non-targetorgans (161±41).

In summary, here we have described for the first time use of a VLA-4 targeted PET imaging probe, ^64^Cu-CB-TE1A1P-LLP2A, to image extra-osseous MM tumors in animal models using small animal PET/CT and tissue biodistribution. Future work will expand the approach to perform longitudinal imaging studies focused on intra-medullary tumor lesions in mouse models of mouse and human MM. Additionally, we will evaluate VLA-4 expression as a marker of effective therapy by molecular imaging, and the results will be compared with the existing imaging standards such as FDG-PET/CT and MRI.

## Supporting Information

Figure S1 **Small animal PET/CT images showing high tumor uptake at early and late time points.** Representative maximum intensity projection (MIP) images of the same mouse bearing matrigel assisted s.c. 5TGM1 tumor in the nape of the neck at 2 h and 24 h post injection. Over time, the tumor to background ratios are improved as the radioactive probe clears out from non-target organs.
(TIF)
