# Correction: Very Late Antigen-4 (α_4_β_1_ Integrin) Targeted PET Imaging of Multiple Myeloma

**DOI:** 10.1371/annotation/8532e291-73d3-461c-810d-0533ccb1eba0

**Published:** 2013-08-23

**Authors:** Deepti Soodgupta, Michelle A. Hurchla, Majiong Jiang, Alexander Zheleznyak, Katherine N. Weilbaecher, Carolyn J. Anderson, Michael H. Tomasson, Monica Shokeen

A funding organization was incorrectly omitted from the Funding Statement. The third sentence of the Funding Statement should read: "MHT is also supported by a Senior Research Award from the Multiple Myeloma Research Foundation (MMRF) and the Barnes Jewish Hospital Foundation." 

