# Supplementary figures and images for: Very Late Antigen-4 (α4β1 Integrin) Targeted PET Imaging of Multiple Myeloma
Source: PLoS One. 2013 Feb 8;8(2):e55841. doi: 10.1371/journal.pone.0055841 (PMC3568146; doi:10.1371/journal.pone.0055841)

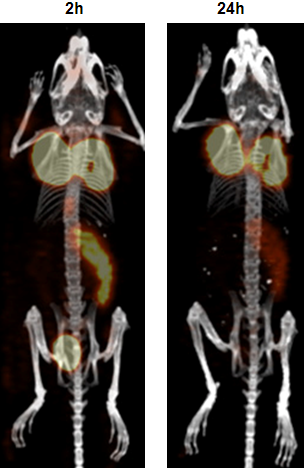

Supplement: Figure S1 — Small animal PET/CT images showing high tumor uptake at early and late time points. Representative maximum intensity projection (MIP) images of the same mouse bearing matrigel assisted s.c. 5TGM1 tumor in the nape of the neck at 2 h and 24 h post injection. Over time, the tumor to background ratios are improved as the radioactive probe clears out from non-target organs. (TIF) [file pone.0055841.s001.tif]
